# Intelligent wide-area resistance surveillance: a novel approach using the semantic web

**DOI:** 10.1186/1753-6561-5-S6-O46

**Published:** 2011-06-29

**Authors:** D Colaert, C Huszka, K Depraetere, H Cools, H Hanberger, C Lovis

**Affiliations:** 1 Advanced Clinical Applications, AGFA Healthcare, Gent, Belgium; 2 Faculty of Health Sciences, Linköping University, Linköping, Sweden; 3 University Hospitals of Geneva, Division of Medical Information Sciences, Geneva, Switzerland

## Introduction / objectives

Proper surveillance of infectious diseases poses special challenges to information technology when it comes to data collection, including wide-area, multi-source and trans-border collection and aggregation of infectious disease and drug resistance information. In this project, we present a novel approach to efficiently monitor bacterial resistance data over multiple international clinical entities.

## Methods

The semantic web provides a common framework that allows data to be shared and reused across applications, beyond the borders of the community and independent of any data source. Our framework is based on multiple international clinical sites within Europe, each of which implementing a site-specific semantic information interface to expose their relevant laboratory data related to antibacterial drug resistance. The data can then be directly queried via a dedicated presentation portal generating summary reports based on various criteria.

## Results

These reports become instantly available on the portal and represent real-time status of drug resistance. They may be used immediately for further processing and decision taking. Rule based alerts may warn operators of unusual patters or happenings. Potential decision support engines may be used to suggest next step scenarios based on information provided.

## Conclusion

We conclude that due to its simplicity, this framework may be easily implemented and maintained with minimal efforts on the information provider’s site paving the way for a secure, real-time site independent data collection. The potential of this framework is immense as the technology itself does not make assumptions on the underlying data provider, practically adaptable to any data source.

## Disclosure of interest

None declared.

